# The Treatment of Movable Kidney

**Published:** 1901-12-21

**Authors:** 


### THE TREATMENT OF MOVABLE KIDNEY.

In many cases of movable kidney, says Mr. Henry Morris, the symptoms—if any—are so slight that no treatment is requisite. When, however, in spite of the avoidance of active exercises, symptoms occur, and when pain, aching, and a wearying sense of dragging weight or of great lassitude are caused by ordinary standing and walking, or when pain occurs even during absolute quietude, nephropexy is called for. It is true that various forms of trusses and bandages have been devised, and have, in some cases, given some partial relief. But though some patients are rendered more or less comfortable by their use, they are not really cured by these appliances. Moreover these pads are not entirely free from risk of doing more harm than good in cases in which the kidney is capable of considerable displacement unless care be taken not to apply them until after the kidney has been replaced in its natural position, or if the kidney descends after the pad has been duly adjusted. In fact, in cases of simple or uncomplicated nephroptosis belts and pads are practically useless, "and some of the frightful things which have been produced under the name of 'kidney pads' ought to be relegated to a chamber for the collection of instruments of torture."

It is chiefly in the rare cases in which movable kidney is associated with enteroptosis that belts are of any real use, and in these cases the best form of belt is one shaped so as to fit well above the hypogastrium, and without any pad, to support the abdomen generally. Many women, however, find a belt so irksome that they positively refuse to continue wearing it, especially when they realise that they can never be cured by its use. Mr. Morris's experience leads him to attach no value to rest in the recumbent position. An operation should certainly be advised when the symptoms are severe and are not relieved by rest, mechanical appliances, and appropriate medicinal and dietetic treatment; when mechanical appliances cannot be borne or seem to increase the symptoms; and when the patient cannot take gentle exercise or even sit erect for long without suffering. In cases in which, in spite of palliative treatment, paroxysms of nephritic colic, fainting, sickness, vomiting, and pain radiating far and wide in the course of the branches of the lumbar plexus occur frequently, the only possibility of relief is surgical operation. Again, the only operation justifiable is nephropexy, which has proved to be safe and successful, and has entirely superseded nephrectomy. As a means of giving relief to the three leading classes of subjective symptoms—namely, pain, gastro-intestinal disturbance, and nervous phenomena—gastropexy has not the same value. It is most useful as a means of relieving pain, less in relieving gastro-intestinal symptoms, and far less in relieving nervous phenomena. Operations upon hysterical patients for the relief of subjective symptoms, even when they are founded upon a real. ascertained physical basis, are not only apt to fail in giving relief, but may arouse ideas which end in fresh complaints or in an aggravation of the original ones. Nevertheless much may depend upon which is the primary affection. If the hysteria or the neurasthenia has been secondary to the movable kidney we should not hesitate to operate, even though the nervous symptoms be pronounced. But we should warn the friends, if not the patient herself, of the speculative nature of the result. The cases in which the operation of nephropexy can be most confidently advised and carried out without any previous trial of belts or of rest, are those of uncomplicated movable kidney in which the principal symptoms are pain and gastro-intestinal disorder, unassociated with general enteroptosis; while the cases in which operation ought to be strongly urged are those in which renal crises are an urgent feature, for in these there is a constant risk of serious injury to the kidney, in the form of hydronephrosis, taking place.

